# The Actual Cautery

**Published:** 1897-11

**Authors:** William Dougherty

**Affiliations:** Baltimore, Md.


### THE ACTUAL CAUTERY.[1]

By William Dougherty, D.V.S.,
BALTIMORE, MD.

It is not my intention to give you the history or the different modes of using the actual cautery, but will confine myself to the use of it in chronic hip- and shoulder-lameness.

Five years ago last March there was sent to me a brown horse lame in the near hip; had been lame for twenty-two months, and had been treated by several practitioners. When the animal arrived at my place he was the lamest horse I had ever seen; the muscles of the gluteal region were so much atrophied that one could see the articulation; he had been blistered several times, also had had setons in hip. The last treatment he had was that of a homœopathic practitioner, and consisted of the administration of internal medicine.

I informed the owner that I did not think it worth while to do anything more, as I would not give any hopes of recovery; but she insisted that I should try, and so I did. Thinking the matter over, it came to my mind that in all the cases of hip-lameness I ever saw recover from the use of a blister there was a slight amount of pus exuded from the joint, and it then occurred to me that the point of the actual cautery would be the proper thing; so I fired him, making five points, viz., one directly over the joint and four surrounding it; on top of that a cantharides oil-blister (one to six) was used.

The result was that there was a free discharge of pus from all five of the punctures; after ten days the animal commenced to im-

[1] Presented at the Thirty-fourth Annual Meeting of the United States Veterinary Medical Association, Nashville, September, 1897.

prove. That consisted of all the treatment, save rubbing the muscles of the hip with white spirits every day. The animal made a com plete recovery by the first of August, and has remained sound to this day (five years and six months).

There were two other cases of hip-lameness, and one of shoulder-lameness, that were nearly as bad as the one I just cited: they also made complete recoveries; the balance of the cases, twenty-eight (making a total of thirty-two), were not so bad.

In some of the milder cases I used but one point of the cautery, directly over the joint, and in all cases using the cantharides oil-blister also. I use a very large and heavy iron with a sharp point, and when the muscles are heavy I fire about one and a half inches deep; when there is much atrophy I do not fire so deep.

I have used, on one or two occasions, the thermo-cautery; that I do not like for this particular operation, as it does not carry fire enough and will cool off in the muscle.

I have also used the large points in cold abscess, fibrous tumors, and in the first stage of fistulous withers; in the fistulous withers I have also used a corrosive-sublimate blister, and had better result than from any other form of treatment.

In this short paper I will not offer any theory, physiology, or pathology of the treatment, and in conclusion will say that I have abandoned the superficial use of the cautery for twenty years—that is, the firing with straight irons, having seen hundreds of cases of superficial firing with no beneficial results, and leaving long cicatrices that were eyesores.

This is a subject that will bear a great deal of thought and investigation, as it is one of the oldest forms of treatment in veterinary practice. I trust that at our next meeting many of our members who are more able and capable will give it ample thought and better elucidate it to the members of this Association.

